# Microparticles in multiple sclerosis and clinically isolated syndrome: effect on endothelial barrier function

**DOI:** 10.1186/1471-2202-15-110

**Published:** 2014-09-22

**Authors:** Beatriz Marcos-Ramiro, Pedro Oliva Nacarino, Esther Serrano-Pertierra, Miguel Ángel Blanco-Gelaz, Babette B Weksler, Ignacio A Romero, Pierre O Couraud, Alberto Tuñón, Carlos López-Larrea, Jaime Millán, Eva Cernuda-Morollón

**Affiliations:** Centro de Biología Molecular Severo Ochoa, CSIC-UAM, C/ Nicolás Cabrera 1, Cantoblanco 28049 Madrid, Spain; Neurology Department, Hospital Universitario Central de Asturias, C/ Aldea Cerdeño, s/n, 33011 Oviedo, Spain; Immunology Department, Hospital Universitario Central de Asturias, Oviedo, Spain; Weill Medical College of Cornell University, New York, NY USA; Department of Life Sciences, The Open University, Milton Keynes, UK; Division of Hematology and Medical Oncology, Institut Cochin, Université Paris Descartes, Paris, France

**Keywords:** Multiple sclerosis, Clinically isolated syndrome, Microparticles, Endothelial barrier function, Thrombin

## Abstract

**Background:**

Cell-derived microparticles are secreted in response to cell damage or dysfunction. Endothelial and platelet dysfunction are thought to contribute to the development of multiple sclerosis (MS). Our aim here is, first, to compare the presence of microparticles of endothelial and platelet origin in plasma from patients with different clinical forms of MS and with clinically isolated syndrome. Second, to investigate the effect of microparticles on endothelial barrier function.

**Results:**

Platelet-poor plasma from 95 patients (12 with clinically isolated syndrome, 51 relapsing-remitting, 23 secondary progressive, 9 primary progressive) and 49 healthy controls were analyzed for the presence of platelet-derived and endothelium-derived microparticles by flow cytometry. The plasma concentration of platelet-derived and endothelium-derived microparticles increased in all clinical forms of MS and in clinically isolated syndrome versus controls. The response of endothelial barriers to purified microparticles was measured by electric cell-substrate impedance sensing. Microparticles from relapsing-remitting MS patients induced, at equivalent concentrations, a stronger disruption of endothelial barriers than those from healthy donors or from patients with clinically isolated syndrome. MS microparticles acted synergistically with the inflammatory mediator thrombin to disrupt the endothelial barrier function.

**Conclusions:**

Plasma microparticles should be considered not only as markers of early stages of MS, but also as pathological factors with the potential to increase endothelial permeability and leukocyte infiltration.

**Electronic supplementary material:**

The online version of this article (doi:10.1186/1471-2202-15-110) contains supplementary material, which is available to authorized users.

## Background

Multiple sclerosis (MS) is an inflammatory neurodegenerative disease of the central nervous system (CNS) that predominantly affects young adults. MS is highly heterogeneous and is considered by some authors to be a conglomerate of neurological syndromes, in which inflammatory damage and demyelination overlap with chronic neurodegeneration. This complexity means that current pharmacological treatments are directed towards modifying the course of the disease, although there is no effective cure for this pathology [1]. Therefore, a better understanding of MS pathogenesis may help to establish new therapeutic strategies. In addition, improvement of early diagnostic tools could help speed up the initiation of MS treatments.

The etiology of MS remains unknown but it is most likely a combination of genetic and environmental factors deregulating the immune response [2]. Vasculature plays a central role in the disease [3–5]. Alteration of endothelial barriers to small molecules and blood cells contribute to the leukocyte infiltration that causes inflammation and demyelination [6–9]. Endothelial permeability in the brain is altered in different clinical forms of MS even during the earlier stages of the disease [3, 8]. On the other hand, chronic activation of platelets is also associated with MS, although their role or the role of the coagulation cascade in this pathology still needs to be clarified [10]. A recent proteomic analysis of active MS lesions confirmed the importance of the coagulation cascade, in general, and of thrombin-mediated signaling, in particular, in the inflammatory progression of this disease [11].

Clinically, MS is classified into relapsing-remitting (RRMS), secondary progressive (SPMS) and primary progressive (PPMS) subtypes. In 85% of patients who develop definitive MS, onset involves an acute or subacute neurological episode affecting the optic nerves, brainstem or the spinal cord, known as clinically isolated syndrome (CIS). Studies of the natural history of CIS patients are heterogeneous in terms of the clinical presentation and the duration of the follow up, but it is commonly accepted that CIS patients are at high risk of developing MS [12].

Microparticles (MPs) are small vesicles released by a variety of cell types in response to inflammatory mediators [13, 14]. These vesicles are able to bind and signal to different cell types through the interaction of proteins exposed in their surface with their cell counter-receptors [13]. MPs have been proposed as markers of a variety of pathological processes such as endothelial dysfunction [15, 16], systemic lupus erythematosus [17], rheumatoid arthritis [17], stroke [18] and thrombosis [19], but their potential role in the progression of these diseases is not fully characterized. An increase in circulating MPs of endothelial origin has been reported in the relapsing phase of patients diagnosed with the RRMS form, which suggests a correlation between MPs and neurological episodes [20]. Platelet-derived MPs have also been detected in RRMS patients [21, 22], but no comparative analysis of MP levels in MS subtypes has been performed to date.

In the present study we present a comprehensive analysis of circulating platelet- and endothelium-derived MPs in the plasma of the different clinical forms of MS. Compared with normal control subjects, we found a significant and comparable increase in all subtypes, including patients with typical CIS and already recovered, or patients in the remission phase of the disease. Interestingly, we found experimental evidence to suggest that plasma MPs induce human endothelial barrier dysfunction and thus may play an active role in MS progression. RRMS MPs had a stronger effect than CIS or control MPs on transendothelial electric resistance (TEER), when analyzed at the same concentration. TEER is inversely proportional to endothelial monolayer permeability, indicating that MP composition and effect on endothelial barrier differ between MS patients and healthy donors. We also report that MS MPs can potentiate the effect on long-term barrier dysfunction of thrombin. Our results indicate that MP generation in plasma is an early and permanent consequence of inflammatory demyelinating events.

## Results

We investigated MPs in platelet-poor plasma (PPP) from 49 healthy volunteers and 95 patients and the possible role of these MPs in endothelial barrier function. The characteristics of controls and patients are summarized in Table 1.

**Table 1:** **Characteristics of controls and patients enrolled in the study**

	Mean age (95% CI)	Number (%) of females
Control (N = 49)	42.70 (23.11-62.95)	26 (53.10)
Patients (N = 95)	44.35 (11.80-73.88)	62 (66.67)
CIS: 12	36.41 (21.69-49.64)	10 (83.33)
RRMS: 51	39.95 (11.80-68.51)	35 (68.63)
SPMS: 23	53.51 (38.38-73.88)	14 (60.87)
PPMS: 9	52.68 (44.06-62.48)	3 (33.33)

### Identification of MPs of platelet and endothelial origin in human plasma

**Figure 1:**
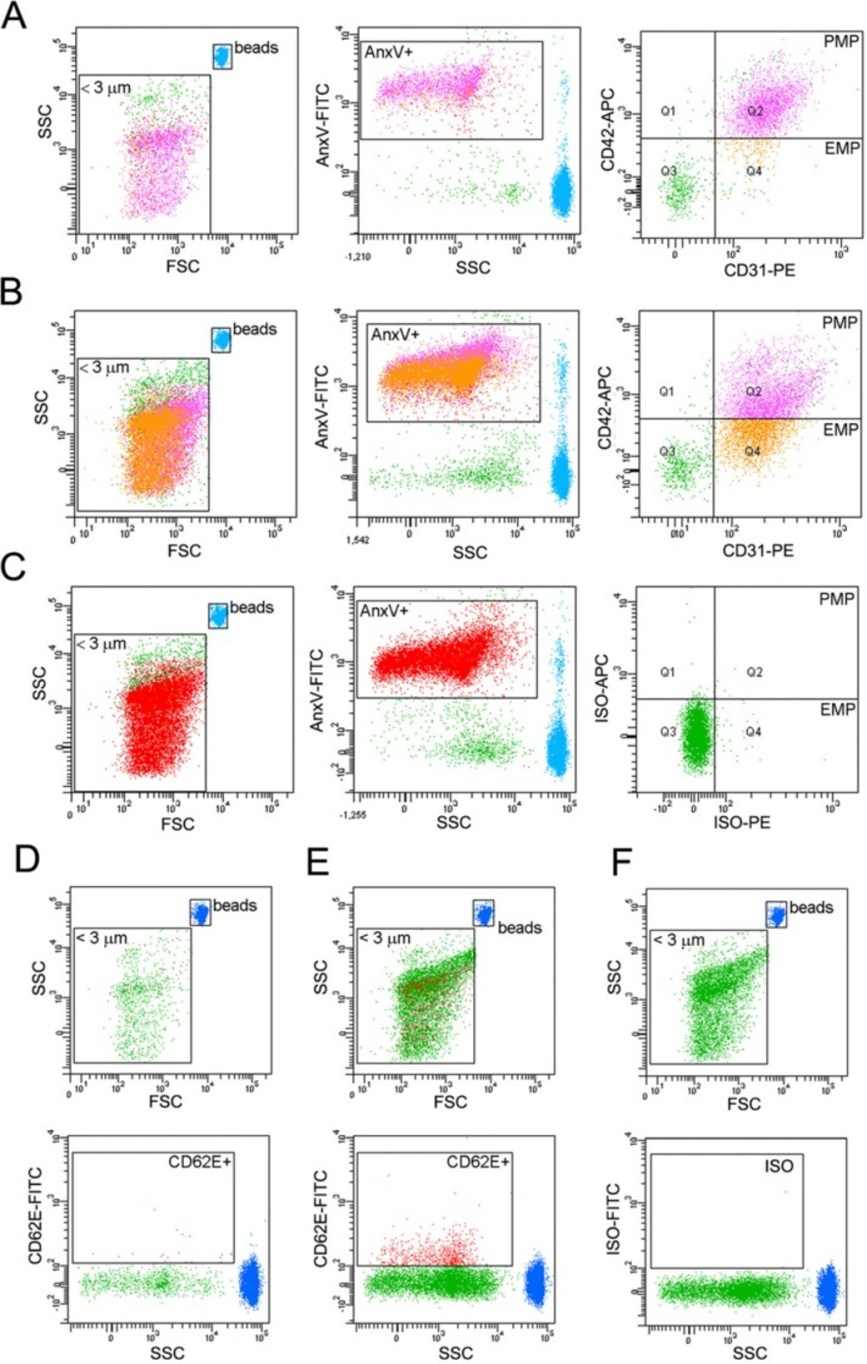
**Detection of PMPs and EMPs in plasma. (A-C)** Flow cytometry scattergraphs for the quantitation of PMPs and EMPs-CD31. MPs smaller than 3 μm were identified by cytometry in the presence of 3 μm diameter beads (left graphs, squared area). These MPs were positive for Annexin V (central graphs, squared areas). Additional incubation with anti-CD31 and CD42 antibodies (**A**, **B**, right graphs) yielded two populations: AnxV^+^ MPs positive for CD31 and CD42 (Q2), which suggests a platelet origin for this MP subpopulation (PMP), and AnxV^+^ MPs, positive for CD31 and negative for CD42 (Q4), which suggests an endothelial origin (EMP). Prior incubation with an antibody isotype control (iso) yielded no positive staining **(C). (A)** Plasma from healthy control, **(B, C)** plasma from multiple sclerosis patient (RR) **(D-F)** Flow cytometry scattergraphs showing the identification of EMPs-CD62. A subset of MPs smaller than 3 μm beads (top graphs, squared area) was positive for an anti-CD62E antibody (CD62E) (**D**, **E**, bottom graphs, squared areas) and negative for an antibody isotype control (iso) (**F**, bottom graph, squared area). **(D)** Plasma from a healthy control **(E, F)** plasma from multiple sclerosis patient (RR).

The PPPs from healthy donors and patients were analyzed by flow cytometry to detect circulating vesicles or MPs of less than 3 μm diameter (Figure 1A-C, left panels, Figure 1D-F, top panels). We found MPs positive for Annexin V, CD42b and CD31 (AnxV + CD42b + CD31+), which suggests a platelet origin, and MPs positive for Annexin V and CD31, but negative for the CD42b marker (AnxV+/CD42b-/CD31+), which suggest an endothelial origin (Figure 1A-B, central and right panels) [20, 23]. Prior cytometer adjustments using isotype specific controls indicated that the signal from these antibodies was specific (Figure 1C, right panels, see Methods). Thus, these two types of MPs were defined as platelet-derived MPs (PMPs) and endothelial-derived MPs (EMPs). MPs positive for the endothelial marker E-Selectin/CD62E + were also found, which confirmed the endothelial origin of an MP subset (Figure 1D-F). Prior cytometer adjustments using isotype specific controls indicated that the signal from anti E-Selectin/CD62E + antibody was specific (Figure 1E,F, bottom panels, see Methods). Hence, EMPs were identified by detecting the markers CD31 and CD42 (EMPs-CD31) or CD62E (EMPs-CD62E).

### Plasma levels of PMPs and EMPs are elevated in patients with CIS and all the clinical forms of MS

**Figure 2:**
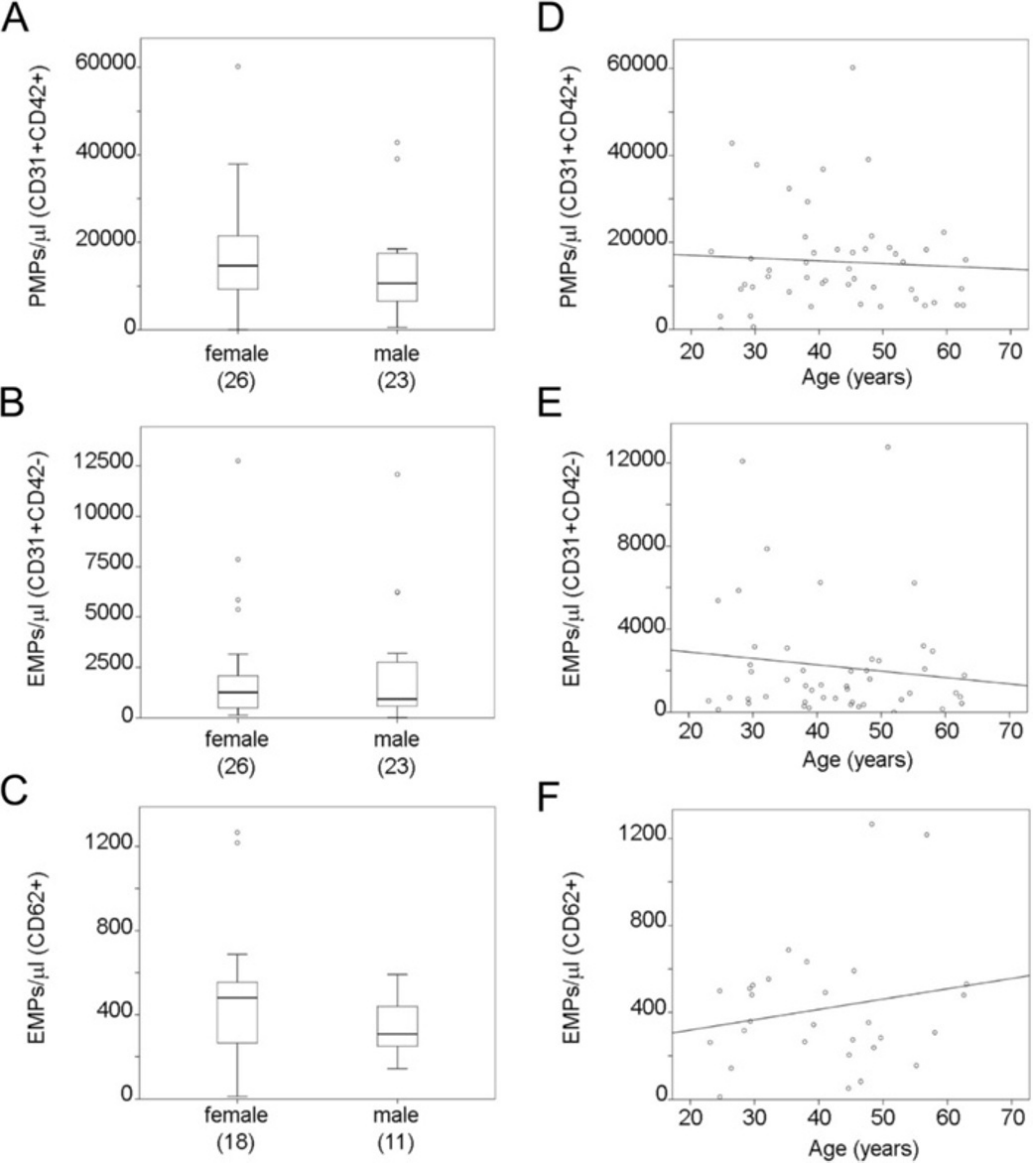
**Gender and age have no effect on PMP and EMP counts in healthy controls.** **(A-C)** Comparison of PMPs **(A)**, EMPs-CD31 **(B)** and EMPs-CD62E **(C)** counts between female and male healthy controls. MPs were identified and quantified by cytometry as in Figure 1. No significant differences were observed (Student’s t-test). **(D-F)** No significant changes in PMPs **(D)**, EMPs-CD31 **(E)** and EMPs-CD62E **(F)** levels were detected in relation to age in healthy donors.

**Figure 3:**
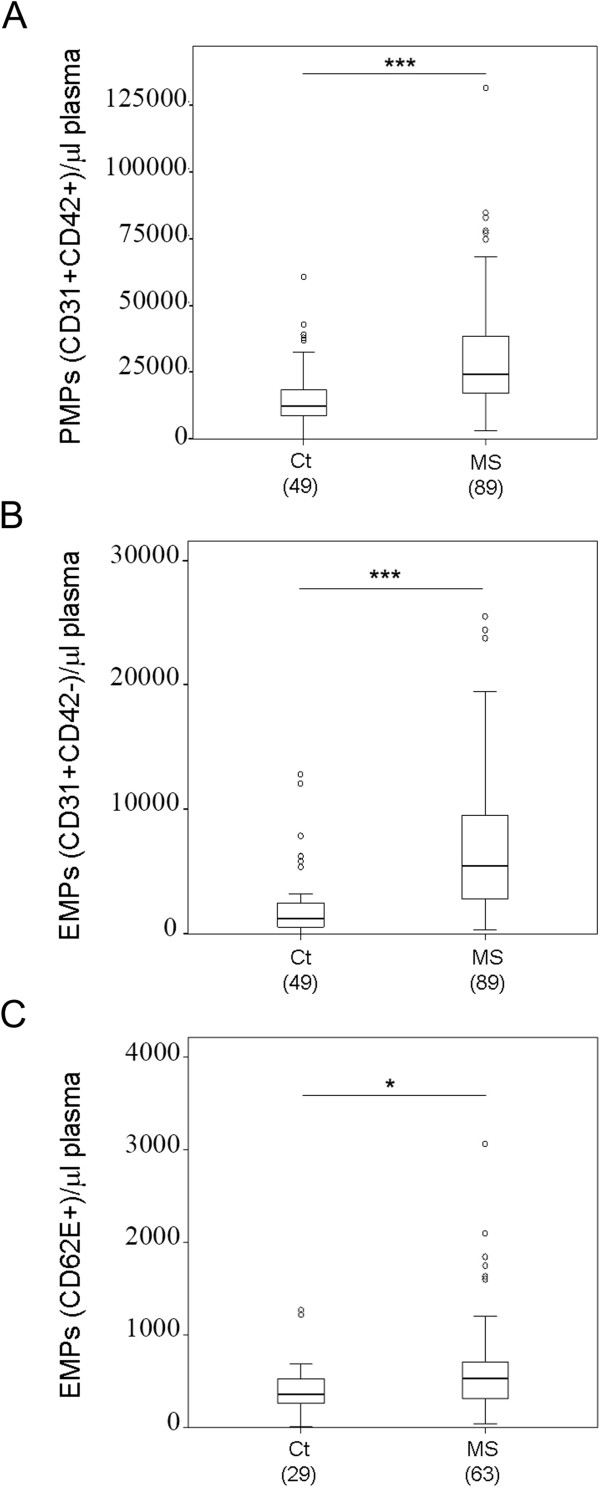
**Circulating MPs are more abundant in MS patients.** Comparison of PMPs **(A)**, EMPs-CD31 **(B)** and EMPs-CD62E **(C)** counts in healthy controls (Ct) and MS patients. MPs were identified and quantified by cytometry as in Figure 1. (***p < 0.001, *p < 0.05 *vs.* healthy controls).

**Figure 4:**
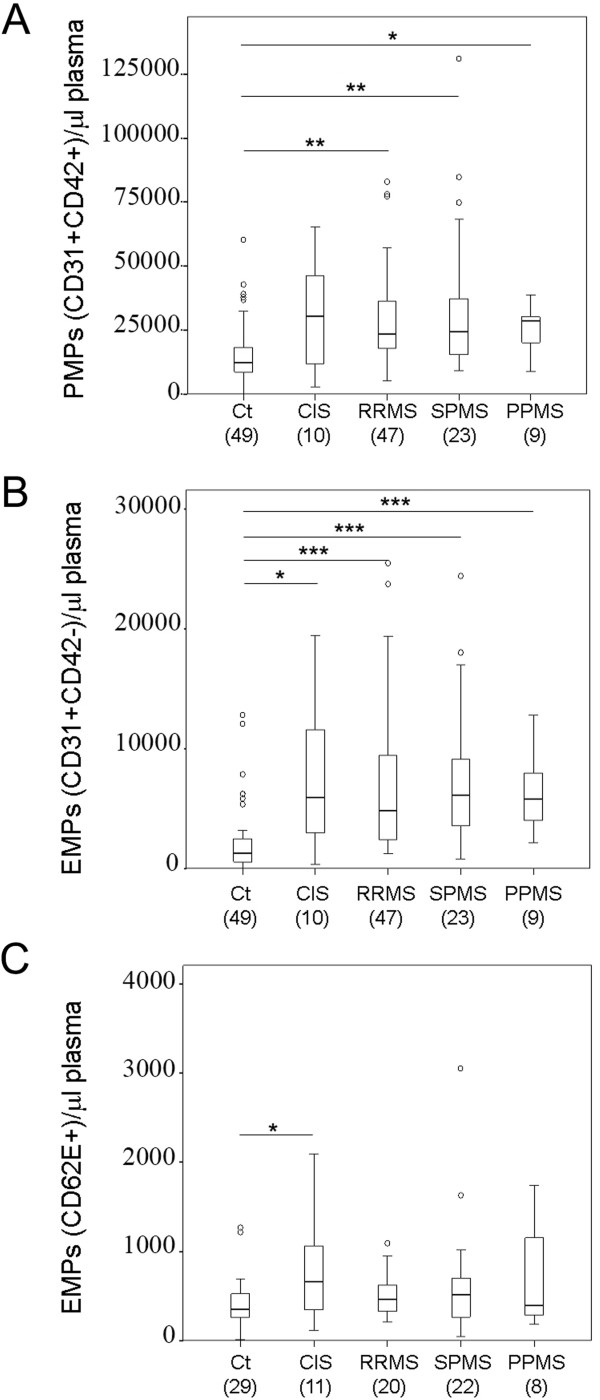
**MPs in the different clinical forms of MS.** Comparison of PMP **(A)**, EMPs-CD31 **(B)** and EMPs-CD62E **(C)** counts in healthy controls (Ct) and patients with CIS or MS. (*p < 0.05, **p < 0.01, ***p < 0.001 vs. healthy controls). CIS, clinically isolated syndrome. RRMS, relapsing, remitting MS. SPMS, secondary progressive MS. PPMS, primary progressive MS. Differences between pairs were assessed by Student’s t-test. MPs concentrations were not able to discriminate between the different clinical forms of MS (ANOVA). Numerical data and results of statistical analysis are shown in Table 2.

Plasma EMPs may reflect age-related endothelial dysfunction [24]. In the healthy donors included in our study, ranging from 24 to 62 years of age, no statistically significant differences in MP number could be attributed to gender or age (Figure 2A-F). This suggests that any change detected in CIS and MS patients cannot be attributed to these parameters. Next, we performed a comparative analysis of circulating MP levels between these control individuals and patients with CIS and all clinical forms of MS. First, the analysis of the pool of all MS patient samples revealed that the mean ± SD of the counts/μl plasma were significantly higher than in healthy controls for the three types of MPs analyzed: 27,203 ± 16,767 for PMPs *vs.* 15,646 ± 11,901 for controls (p < 0.001) (Figure 1A,B; Figure 3A) 6,527 ± 4,554 EMPs-CD31 *vs.* 2,202 ± 2,783 for controls (p < 0.001) (Figure 1A,B; Figure 3B), and 746 ± 642 for EMPs-CD62E *vs.* 418 ± 289 for controls (p < 0.05) (Figure 1D, E; Figure 3C). An elevated MP content was also detected when each clinical form of MS was individually analyzed, including the progressive forms, SPMS and PPMS, which are considered to have a less important inflammatory component (Figure 4). The MP counts (mean ± SD) for each form of MS is summarized in Table 2. PMPs were higher than controls in CIS and all the MS forms, but the increase was not statistically significant for CIS patients (Figure 4A, Table 2). In addition, remarkable and statistically significantly higher levels of EMPs-CD31 were observed in samples from CIS and all MS forms compared to control donors (Figure 4B, Table 2). Finally, compared to control subjects, EMPs-CD62 were augmented in CIS and all the MS forms, although this increase was statistically significant only for CIS patients: 646 ± 195 *vs.* 418 ± 289 (p < 0.05) (Figure 4C). In summary and regarding the absolute values of MP counts, our results show that patients with CIS and all the clinical forms of MS have comparable levels of circulating MPs in plasma, which are higher than those in healthy individuals (Table 2).

**Table 2:** **Circulating MP counts in healthy controls and the different clinical forms of MS**

	PMPs mean (SD) counts/μl	EMPs-CD31 mean (SD) counts/μl	EMPs-CD62 mean (SD) counts/μl	PMPs p ***vs.***Ct	EMPs-CD31 p ***vs.***Ct	EMPs-CD62 p ***vs.***Ct
Control	15,646 (11,901)	2,202 (2,784)	418 (289)			
CIS	30,936 (22,550)	7,964 (6,888)	646 (195)	n.s.	<0.05	<0.05
RRMS	28,929 (18,247)	7,136 (6,088)	511 (231)	<0.001	<0.001	n.s.
SPMS	34,188 (29,511)	7,512 (5,962)	629 (644)	<0.01	<0.001	n.s.
PPMMS	26,422 (9,865)	6,460 (3,610)	699 (621)	<0.05	<0.001	n.s.

(n.s.: not statistically significant). p values compared to Control group, Student’s t-test.

### MPs induce endothelial barrier dysfunction

**Figure 5:**
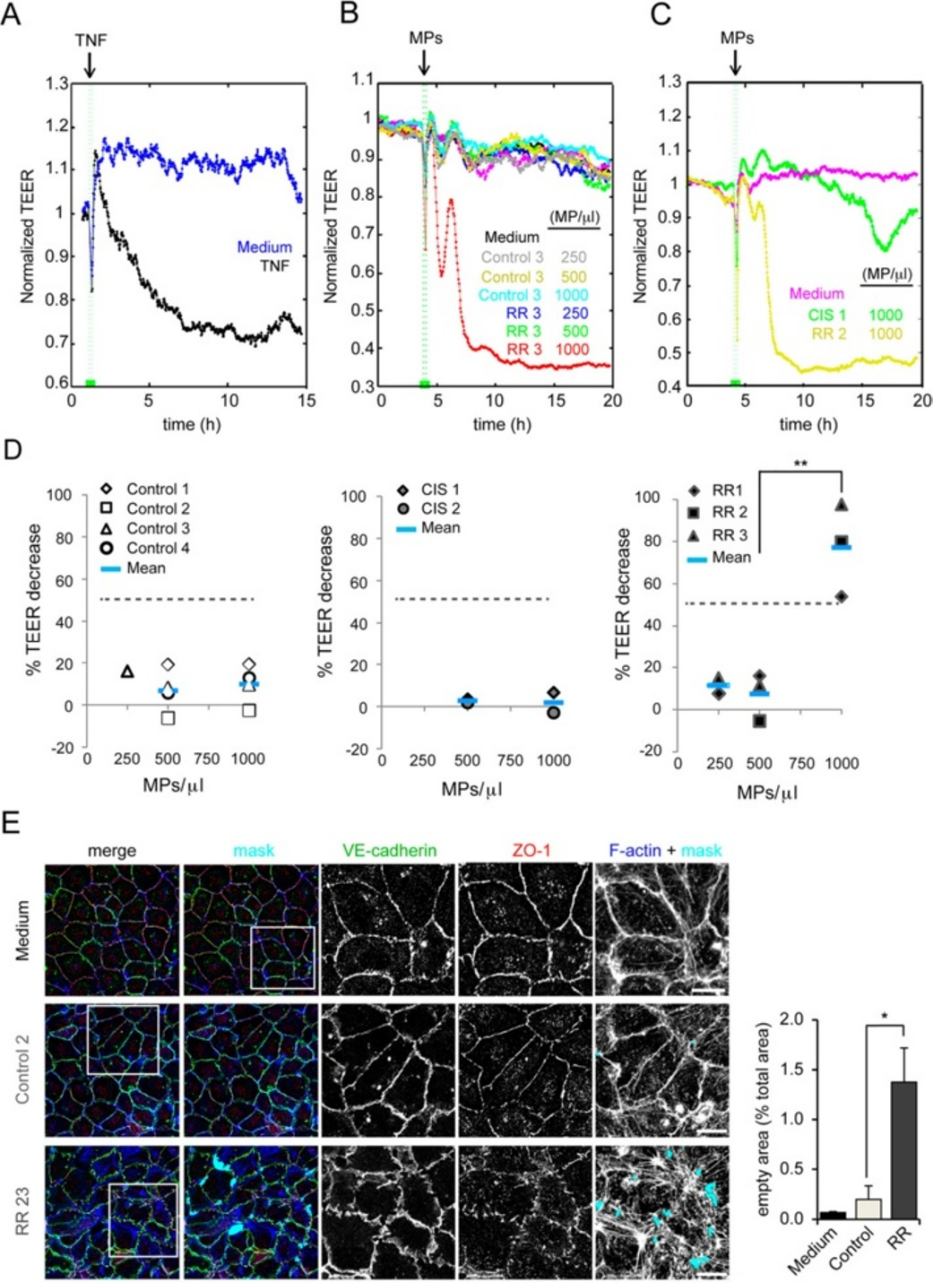
**Effect of MPs on HUVEC barrier function. (A)** TNF is a paradigmatic stimulus that induces significant and progressive reduction of TEER (normalized TEER) **(B, C)** Normalized TEER of confluent HUVECs left untreated (Medium) or exposed to MPs at the indicated concentrations. MPs from one healthy control (Control 3) and one RRMS patient (RR 3) were compared in a **(B)** and MPs from one CIS patient and from a RRMS patient (RR 2) were compared in **(C)**. **(** **D** **)** Percentage of TEER decrease induced after 14 hours of incubation with Control, CIS and RR-MPs at 250, 500 and 1000 MP/μl (see Methods). Average TEER decrease in response to TNF is marked by discontinuous lines. **p = 0.004. **(E)** HUVECs left untreated (Medium) of previously treated for 14 h with MPs from a healthy control and a RRMS (RR) patient at 1000 MP/μl were stained for the cell-cell junction markers VE-cadherin and ZO-1 and for filamentous actin (F-actin). Semi-automated image processing identified intercellular gaps in the images (mask) that were quantified respect to the total area of the cell monolayer (right graph). *p = 0.01. Bar, 20 μm.

**Figure 6:**
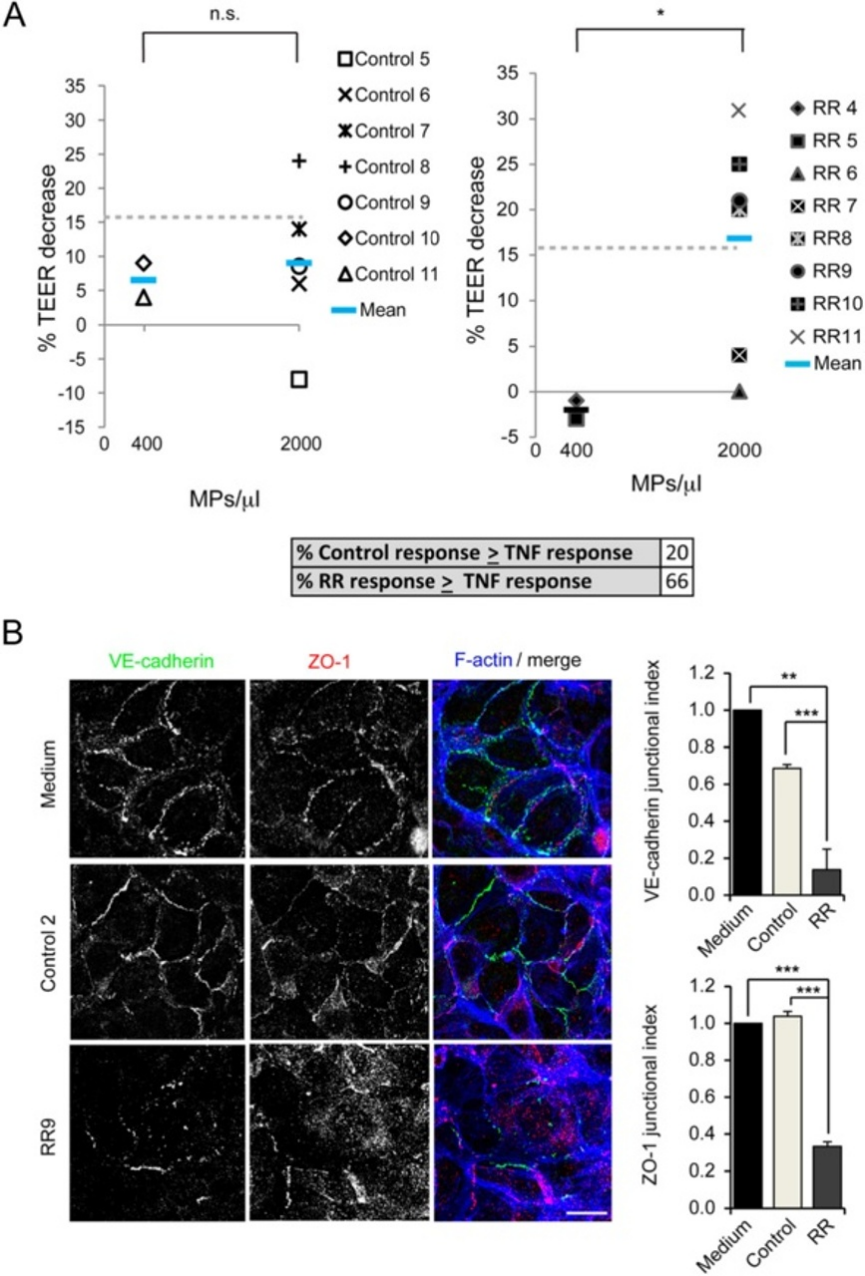
**Effect of MPs on hCMEC/D3 barrier function. (A)** Percentage of TEER decrease after 14 hours of incubation with the indicated MPs at 400 and 2000 MP/μl. Left graph, control donors. Right graph, RRMS patients (RR). Average TEER decrease in response to TNF is marked by discontinuous lines. Bottom table shows the percentage of MPs inducing a response stronger than control TNF on the endothelial barrier. *p = 0.04. **(B)** VE-cadherin, ZO-1 and F-actin staining in HCMEC/D3 cells exposed for 14 h to MPs from a donor and a RRMS patient. MPs did not induced big gaps in HCMEC/D3, as in HUVECs, but, instead, RR-MPs dispersed the junctional staining of VE-cadherin (top right graph) and ZO-1 (bottom right graph) quantified as the ratio between the staining intensity at cell-cell borders and at the inner cell area (Junctional index). **p < 0.03, ***p < 0.002. Bar, 20 μm.

Endothelial barrier dysfunction is a hallmark of MS. To gain insight into the role of circulating MPs in MS we compared the effect on endothelial barrier function of MPs isolated from patients and healthy controls. We used an electric cell-substrate impedance sensing (ECIS) system that measures in real time the resistance of endothelial monolayers to a weak electric current that cause no effects on cells. This is called transendothelial electric resistance (TEER) and is inversely proportional to the permeability of the monolayer. To address the relevance of MP-mediated TEER changes in each endothelial cell type, cells were incubated in parallel with the inflammatory cytokine tumor necrosis factor (TNF) as a positive control of *in vitro* barrier disruption (Figure 5A) [25, 26]. First, monolayers of human umbilical vein endothelial cells (HUVECs) were incubated with growing concentrations of MPs from healthy donors, CIS patients and RRMS (RR-MPs) patients, the latter taken as paradigm of patients in which the disease has already progressed (Figure 5B and C). Control, CIS and RR-MPs had no significant effects on constitutive TEER at concentrations of 250 and 500 MP/μl. In contrast, MPs from RRMS patients notably disrupted the endothelial barrier after 4 h of incubation at 1,000 MP/μl (Figure 5B-D; Additional file 1: Figure S1A). These MPs decreased normalized TEER by 77.14 ± 21.94%, (p = 0.004) (Figure 5D). Such decrease was expressed as the percentage of the difference between the TEER values obtained from unstimulated HUVEC monolayers (before incubation with MPs) and the TEER values measured in absence of cells (see Methods). In addition, this decrease was comparable or even stronger than that caused by TNF exposure (Figure 5A and 5D, discontinuous line). In contrast, barrier alterations that control and CIS MPs induced on endothelial barrier function at 1,000 MP/ml were clearly below the effect of TNF and were considered minor (Figure 5A-D). Consistent with the loss of TEER upon exposure to MPs from RRMS patients, the confocal analysis of endothelial cells incubated with these MPs showed the appearance of intercellular gaps, detected by staining of F-actin and the junctional markers VE-cadherin and ZO-1 (Figure 5E, mask, see Methods). In contrast, intercellular gaps were absent or rare in cells incubated with control and CIS MPs. These intercellular gaps were measured as the percentage of empty spaces found in different regions of the cell monolayer and increased from 0.20 ± 0.23% in cells exposed to control MPs to 1.38 ± 0.59% in cells exposed to RR-MPs (p < 0.02) (Figure 5E). Together, these data suggest that MPs from RRMS patients have composition and signaling properties different to control and CIS MPs. To confirm this, we tested the effect of RR-MPs in a cell model of human endothelium from the blood–brain barrier (BBB), the HCMEC/D3 cells [27]. We found no effect of MPs on HCMEC/D3 monolayers at concentrations of 400 (Figure 6A) and 1,000 MP/μl (not shown). However, barrier disruption caused by RR-MPs at 2000 MP/μl was stronger than that produced by TNF or by control MPs at the same concentration (Figure 6A, Additional file 1: Figure S1B). In the presence of RR-MPs, resistance decreased by 16.83 ± 12.19% (p < 0.04), whereas in the presence of 2,000 MP/μl of control MPs, the resistance decreased only by 9.00 + 11.7% (p = 0.39), which was no statistically significant. 66% of RR-MPs had an effect higher than TNF versus only 20% for control MPs (Figure 6A). In contrast with the effect observed in HUVECs, the incubation with RR-MPs did not induce big intercellular gaps in HCMEC/D3 cells, but caused a significant decrease of the immunofluorescence staining of VE-cadherin and ZO-1 at cell-cell junctions (Figure 6B). This was expressed as the junctional index, in which the ratio between the staining intensity at cell borders and the staining at the cell inner area was measured per cell in confluent cell monolayers. This ratio was normalized to 1 for HCMEC/D3 that had not been exposed to MPs (see Methods). Junctional index significantly decreased only in the presence of RR-MPs. It was reduced to 0.14 ± 0.06 (p < 0.03) for VE-cadherin staining and to 0.33 + 0.03 (p < 0.002) for ZO-1 staining. Thus, healthy donors not only have significantly less MPs in plasma than MS-patients. At equal concentrations, MPs from RRMS patients provoke higher disruption of endothelial barrier properties that those from healthy donors.

**Figure 7:**
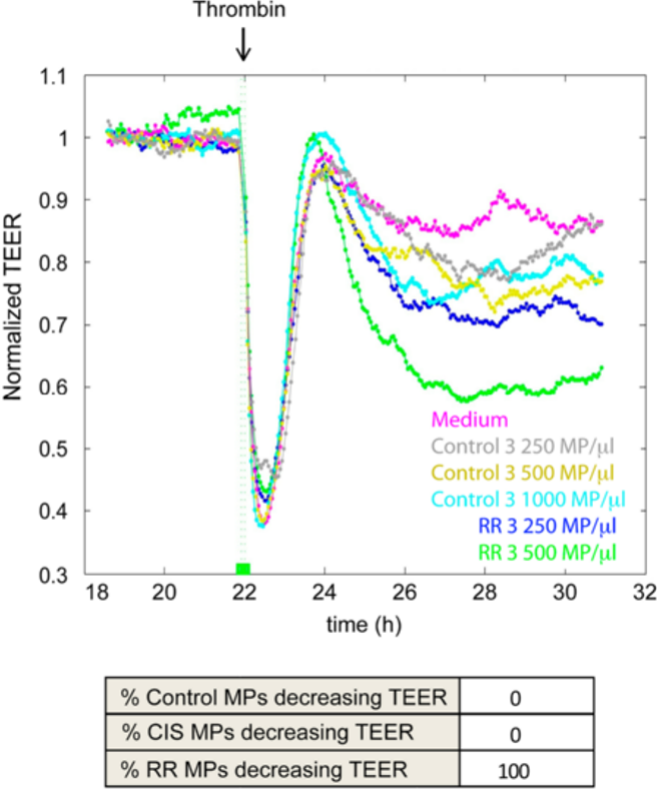
**Synergistic effect of MS-MPs and thrombin on endothelial barrier function.** Top graph. Representative experiment in which HUVECs were incubated with MPs at concentrations between 250 and 1000 MP/μl for 22 h. Then, 1 U/ml of thrombin was added to those monolayers showing no alteration of TEER in response to MPs. After thrombin-induced acute contraction and subsequent barrier recovery, HUVECs preincubated with RRMS MPs (RR3) undergo long-term decrease in TEER. Bottom table. Percentage of Control (3 samples), CIS (2 samples), and RR-MPs (3 samples) in which 500 MP/μl had no apparent effect on barrier function, but induced long-term TEER decrease after thrombin stimulation.

The importance of thrombin in the inflammatory progression of experimental MS has recently been shown [11]. Thrombin is an inflammatory mediator that induces acute barrier contraction and subsequent long-term inflammatory activation of the endothelium [28], so we hypothesized that MPs with no apparent effect on endothelial barriers on their own, may sensitize cells to thrombin-mediated barrier disruption. Twenty-two hours after the addition of MPs, the endothelial responses to thrombin were analyzed in HUVECs and HCMEC/D3 monolayers in which TEER had not been previously altered by the initial incubation with MPs. Whereas HUVEC monolayers were transiently but completely disrupted by thrombin (Figure 7), we unexpectedly found that the HCMEC/D3 cell line barely contracted in response to this inflammatory mediator (not shown). This suggests that transformed HCMEC/D3 cells may lack some protein machinery important for a full response to thrombin. We thus studied the effect of MPs and thrombin only in HUVEC barriers. The acute phase of contraction upon thrombin stimulation and the subsequent TEER recovery were not affected by the presence of MPs in these endothelial cells (Figure 7). However, between 3 and 8 h after thrombin stimulation, cells initially exposed to RR-MPs at 500 MP/μl gradually reduced their barrier integrity. In contrast, the TEER decrease between 3 and 8 h after thrombin activation was transient and minor in cells previously exposed to control or CIS MPs (Figure 7). Together these data suggest that MPs have an effect on endothelial barrier function either on their own, at higher concentrations, or when acting synergistically at lower concentrations with a proinflammatory stimulus, namely thrombin, important for MS progression. Together, these data suggest that chronic exposure to MPs may contribute to a long-term increase in extravasation of molecules and cells from the bloodstream in MS patients. Further investigation into the protein composition of MPs from different clinical subtypes may help the design of therapies in which the endothelial permeability increase associated with this inflammatory disease could be prevented.

## Discussion

In this study we show that the different clinical forms of MS, including the progressive forms, are associated with platelet and endothelial dysfunction, as determined by an increase in the number of circulating platelet- and endothelial cell-derived MPs [21, 29]. Moreover, we demonstrate that these vesicles may play an active role in the progression of the disease by increasing endothelial monolayer permeability.

### Circulating microparticles as potential markers of CIS and MS

Different soluble markers have been described for MS in plasma or cerebrospinal fluid [30–32]. Among others, platelet endothelial cell adhesion molecule (PECAM)-1 (CD31) and E-Selectin (CD62E) are present in microvesicles derived from endothelial cells during apoptosis or upon inflammatory stimulation [29] and their concentration in plasma may account for the status of the endothelium [33]. In our study we observed a remarkable increase in PMPs and both EMPs in all MS clinical forms. The fact that elevated levels of EMPs in CIS patients were also found indicates that these circulating vesicles are a chronic and early feature in patients experiencing proinflammatory demyelinating pathologies. Our results extend and are partly consistent with a previous report showing an increase in EMPs, defined as CD51+ in both the exacerbation and remission phases of RRMS patients. Interestingly, these authors find no differences in CD31+/CD42b- MPs in patients during the remission phase, although they elegantly show that isolated endothelia from patients in both the exacerbation and remission phases of the disease release similar levels of both CD31+ and CD51+ MPs to plasma, which are higher than those released by the endothelium of healthy controls [20]. Collectively and in line with our results, this work indicates that in the remission phase of RRMS, high levels of EMPs can be found. However, MPs from the exacerbation and remission phases of the disease probably differ in composition. On the other hand, we have found that plasma PMPs were significantly increased in each MS subtype, consistent with the elevated circulating PMPs previously found in RRMS. These data are also consistent with a pivotal role for platelets in MS [21, 34]. PMPs were also elevated in CIS patients, but this increase was not statistically significant. This suggests that platelet dysfunction may occurs later than the endothelial dysfunction and when the disease is definitively progressing. Indeed, platelets have been found in human MS lesions and in the CNS of mice in the EAE model. Platelet depletion in the EAE model ameliorated the disease, which was associated with a reduction in recruitment of leukocytes to the CNS. Similar results were observed after treatment with an anti-CD42b antibody. It is interesting to note that CD42b is present at the PMP surface and therefore the potential role of these vesicles in the progression of the disease needs to be considered.

Signals that impair BBB function in MS are initially originated in the central nervous system. It is of note that in the early stages of this pathology, the microglia releases reactive oxygen species, TNF and IFN-γ, all of which can induce MP release [35]. Therefore, the initial inflammatory foci initiating the progression of the disease may cause the early secretion of EMP. In summary, we have observed an increase in circulating EMPs both in CIS and the remitting phase of RRMS, suggesting enduring endothelial dysfunction from the very early stages of demyelinating pathologies. These results make PMPs and EMPs good candidates for clinical markers to identify and discriminate between CIS and early MS.

### Circulating microparticles as active players in MS progression

The remarkable ability of MPs to induce cell signaling and to promote endothelial dysfunction has already been reported [36]. PMPs may activate leukocytes and induce their transendothelial migration [37]. Moreover, MPs promote a procoagulant status due to the presence of phosphatidylserine in the outer part of the membrane [38]. All this evidence suggests a potential role for MPs in the progression of different pathologies, including MS.

The endothelial beds are highly exposed to circulating MPs and therefore, more likely to respond to MP-mediated signaling. The data presented here demonstrate that MPs alone increase endothelial permeability. Moreover, this effect differs between definitive MS, stronger, and CIS patient and control donors, which are more attenuated when compared at the same concentration, suggesting differences in the composition of these microvesicles. Various mechanisms could mediate the effect of MPs on endothelial barrier function. MPs expose surface receptors with the potential to regulate endothelial permeability. The marker CD31, used for the identification of EMPs, can establish homotypic interactions and modulate endothelial permeability [26]. EMPs also regulate the production of reactive oxygen species (ROS) [39]. ROS increase causes disruption of the endothelial barriers. On the other hand, MPs from microvascular endothelial cells and from atherosclerotic plaques contain matrix metalloproteinases involved in the cleavage and shedding of surface proteins, including that of TNF [40, 41]. Thus, MPs may contribute to the release of cytokines that locally increase permeability. Indeed, MPs have been shown to act as proinflammatory agents. MPs from monocytes contain inflammatory cytokines with the potential to modulate permeability and components of the inflammasome. These MPs activate the transcription factor NF-κB and induce the expression of adhesion receptors in the endothelium [42]. Finally, MPs also transport RNA and micro (mi) RNA, which have the potential to modulate protein expression in the target cell [43]. Some miRNAs, such as miRNA155 negatively affects BBB function during neuroinflammation [44]. Hence, the analysis of the different composition of healthy and MS microparticles could reveal novel targets for preventing endothelial barrier disruption during the progression of the disease.

MS is considered to be a chronic inflammatory disease in which several inflammatory mediators play a relevant long-term function in its progression. Permanent exposure to these mediators is probably the origin of the MP increase but, importantly, it may also amplify the effect of MPs on the endothelium. In the EAE model, it has been reported that thrombin inhibition ameliorates the neurological symptoms, indicating a deleterious effect of this mediator in the exacerbation phase [11]. In MS, various proteins involved in coagulation, including tissue factor, which activates thrombin, become more abundant in chronic active plaques [11]. Thrombin is therefore a cytokine at the crossroads of inflammation and coagulation with a remarkable ability to signal to and to alter the barrier properties of endothelial cells, thereby possibly contributing to the progress of MS. Our data indicate that preincubation with RR-MPs has a long-term effect on the endothelial barrier response to thrombin-mediated challenge, which induces a secondary decrease in TEER between 3 and 8 h post-stimulation. As mentioned above, MPs can deliver proinflammatory molecules. This synergic effect on thrombin-mediated signaling occurs within a time frame compatible with the modulation of the expression of genes related to inflammation and barrier function [28] rather than an effect on acute actomyosin-mediated contraction [45]. Therefore, MS-MPs may alter the vasculature on their own, or potentiate the effects of proinflammatory mediators on endothelial barrier dysfunction. This may have important and unexplored consequences for the progression of MS. Further studies are necessary to determine which components of MS-MPs are responsible for the observed effects.

## Conclusions

Our findings demonstrate that platelet and endothelial functions are altered in the different clinical forms of MS, since patients show an increase in circulating MPs of endothelial and platelet origin. In addition, endothelial MPs are also significantly increased in CIS patients. *In vitro*, MPs from MS patients disrupt endothelial barriers and may thus cooperate in the progression of the disease.

## Methods

### Patients and controls

Eighty-three adult MS patients who met the criteria of Poser [22] and MacDonald [46], 12 adult patients with a typical CIS, suggestive of MS, and 49 healthy adult volunteers gave their written informed consent and were enrolled in the study, which was approved by the Ethics Committee of the Hospital Universitario Central de Asturias (Oviedo). MS exacerbation was defined as a worsening of neurological impairment or the appearance of a new symptom attributable to MS and lasting for at least 24 hours. Patients were exacerbation-free and none had any corticosteroids for at least one month before entering the study.

### Sample collection

Venous blood was collected in citrate vacutainer tubes with a 21G needle. Blood was centrifuged within 20 min of extraction. PPP was obtained by centrifugation for 20 min at 1,550 *g*. Aliquots were immediately frozen and preserved at -80°C until use.

### Antibodies and chemicals

PE-conjugated anti-CD31 was from eBioscience (San Diego, CA, USA). APC-conjugated anti-CD42b was purchased from BD Bioscience (Erembodegem, Belgium). FITC-conjugated anti-CD62E (E-Selectin) was obtained from Santa Cruz Biotech (Santa Cruz, CA, USA). FITC-conjugated Annexin V was from ImmunoStep (Salamanca, Spain). Recombinant fibronectin, thrombin and other chemicals were provided by Sigma-Aldrich (St Louis, MO, USA).

### Flow cytometry

A volume of 20 μl of PPP was incubated with 2 μl of the indicated antibodies or their corresponding isotypic controls at room temperature for 20 min with gentle shaking (100 rpm). Following this, 900 μl of PBS containing a fixed number of 3-μm latex beads were added. For the AnxV + MP determination, 4 μl of FITC-conjugated AnxV were added with the antibodies and the sample was subsequently diluted in 900 μl of AnxV buffer containing the latex beads. MPs present in the samples were measured in a FACSAria cytometer with the FACSDiva software (BD Bioscience, Erembodegem, Belgium). To determine the fluorescence background, isotypic antibodies for each fluorochrome were used. Compensation adjustments were made based on fluorescence minus one (FMO) controls, which consist of all the reagents but the one of interest. The absolute number of MPs was estimated through the formula: [MP/μl plasma] = (n° events counted per test * n° beads per test)/(n° events in bead region * test volume). 10,000 beads were routinely collected. All solvents were 0.22-μm filtered.

### Cell culture

HUVECs were purchased from Lonza (Barcelona, Spain) and grown in fibronectin-coated plates in EBM-2 medium supplemented with 2% fetal bovine serum, glutamine, penicillin/streptomycin and the endothelial cell growth supplement EGM-2 [26]. Immortalized hCMEC/D3 cells were obtained as previously described [27] and grown in rat collagen-I-coated plates (Cultrex) in EBM-2 medium supplemented with 5% fetal bovine serum, penicillin/streptomycin, hydrocortisone (Sigma), ascorbic acid (Sigma), Chemically Defined Lipid Concentrate (Invitrogen), HEPES (PAA The Cell Culture Company) and human bFGF (Sigma).

### Isolation of MPs

PPP from patients and controls was centrifuged at 3,200 *g* for 30 min and subsequently at 13,000 *g* for 10 min to remove cell debris. Supernatants were then centrifuged at 18,000 *g* for 45 min and the pellet of MPs washed and resuspended in EC culture medium. Final MP counts were determined by flow cytometry.

### Endothelial permeability assays

Cells were grown to confluency on fibronectin-coated (HUVECs) or rat-collagen-I coated (hCMEC/D3) eight-well array culture-ware (8WE10, Ibidi, München, Germany) specific for transendothelial electric resistance (TEER) measurements with the electric cell substrate impedance sensing (ECIS) system 1600R (Applied Biophysics [26]. The experiments were performed in wells in which the electric resistance of the EC monolayer, which is inversely proportional to its permeability, had reached a steady-state. EC monolayers were incubated with MPs isolated from controls and patients and the effect on permeability monitored by at least 14 h. The percentage of maximum permeability increase induced by MPs was calculated taking into account that the average normalized resistance (NR) for an ECIS electrode containing no cells or fully contracted cells (maximum permeability) is 0.35, whereas untreated, control confluent monolayers yielded an average NR value of approximately 1.10 for hCMEC/D3 and 0.95-1.00 for HUVECs 14 h after the beginning of the experiment. Thus, the percentage of reduction in resistance was calculated applying the formula [(NR unstimulated cells-NR MPs)/0.75] X100, NR being the normalized resistance value 14 h after the beginning of the ECIS reading. A parallel incubation with 10 ng/ml human TNF (R & D) was performed to measure the responsiveness of hCMEC/D3 and HUVEC barriers to inflammatory challenges. 22 h after exposure to the indicated MPs, the ECIS was paused, 1U/ml of human thrombin was added and its effect recorded in the instrument for an additional period of 10 h.

### Immunofluorescence analysis

Endothelial cells were grown at confluency for 48 h in Ibidi μ-slide 8 well dishes pre-coated with fibronectin (HUVECs) or rat-collagen-I (hCMEC/D3). Cells were incubated with MPs from control donors or patients at the indicated concentrations for at least 14 h. In parallel, ECIS assays were performed with the same MPs to detect changes in endothelial barrier function. Then, cells were fixed in 4% paraformaldehyde for 20 min, rinsed and treated with 10 mM glycine for 5 min to quench the aldehyde groups. The cells were then permeabilized with 0.2% Triton X-100, rinsed and incubated with 3% bovine serum albumin in PBS for 15 min. Cells were incubated for 30 min with the indicated antibodies at 37°C, rinsed in PBS and incubated for 30 min with the appropriate fluorescent secondary antibodies. Actin filaments were detected with fluorescent phalloidin. Confocal laser scanning microscopy was carried out using a Zeiss LSM 510 microscope equipped with a 63 × 1.3 NA oil immersion objective.

Intercellular gaps in confluent monolayers were quantified using Image J. Ten images containing around twenty cells were quantified per condition and experiment in three different experiments. The image contrast was semi-automatically increased to saturation, so regions in the confluent monolayer that yielded no signal in all the fluorescence channels were taken as empty areas or intercellular gaps, and selected by creating a threshold. The percentage of empty areas respect to total image area was calculated. To show the empty areas, the region obtained with the threshold was blue-colored and flattened to the original image.

When intercellular gaps were not big enough to be detected, the junctional index was calculated. The junctional index quantified the junctional/non-junctional staining ratio for junctional proteins and was also calculated using Image J. Ten images containing around twenty cells were quantified per condition and experiment. The background was substracted using the *BG Substraction from ROI* pluging from Image J. A region that selected the total area of a single cell in the confluent monolayer was created. This initial region was made 5 to 10 pixels smaller using *enlarge* tool. The intensity of this smaller region was quantified as non-junctional intensity of VE-cadherin or ZO-1. The area between the initial region and the smaller region was considered as junctional. The junctional index was normalized taking as 1 the ratio from cells not exposed to MPs.

### Statistical analysis

Statistical analyses were performed using SPSS 15.0 for Windows. One-way ANOVA (analysis of variance) was used to analyze differences among three or more groups. Pairs of groups were compared using Student’s t test (parametric data) or the Mann Whitney U test (nonparametric data). Bivariate correlations were estimated by Spearman’s rank correlation (R). All tests for statistical significance were two-tailed and values of *p* < 0.05 were considered statistically significant.

## Electronic supplementary material

Additional file 1: Figure S1: Absolute values of TEER detected in endothelial cell monolayers exposed for 14 h to MPs from healthy controls and patients. **(** **A** **)** TEER of HUVECs incubated with 1000 MP/μl from healthy controls, CIS and RRMS (RR) patients (see Figure 5D). Red line marks the average resistance detected in empty electrodes. **(** **B** **)** TEER of HCMEC/D3 incubated with 2000 MP/μl from healthy controls, and RRMS patients (see Figure 6B). Discontinuous line marks the average resistance of TNF-stimulated cells. (PDF 269 KB)

## Abbreviations

MS: Multiple sclerosis
RRMS: Relapsing, remitting MS
SPMS: Secondary progressive MS
PPMS: Primary progressive MS
CIS: Clinically isolated syndrome
CNS: Central nervous system
BBB: Blood–brain barrier
PPP: Platelet-poor plasma
MPs: Microparticles
PMPs: Platelet-derived MPs
EMPs: Endothelial-derived microparticles
EC: Endothelial cell
HUVEC: Human umbilical vein endothelial cell
TNF: Tumor necrosis factor
TEER: Transendothelial electric resistance
ECIS: Electric cell-substrate impedance sensing
AnxV: Annexin V
SD: Standard deviation.
