# Supplementary material for: Microparticles in multiple sclerosis and clinically isolated syndrome: effect on endothelial barrier function
Source: BMC Neurosci. 2014 Sep 22;15:110. doi: 10.1186/1471-2202-15-110 (PMC4261570; doi:10.1186/1471-2202-15-110)
Supplement: Supplementary file 1 — Additional file 1: Figure S1: Absolute values of TEER detected in endothelial cell monolayers exposed for 14 h to MPs from healthy controls and patients. ( A ) TEER of HUVECs incubated with 1000 MP/μl from healthy controls, CIS and RRMS (RR) patients (see Figure 5D). Red line marks the average resistance detected in empty electrodes. ( B ) TEER of HCMEC/D3 incubated with 2000 MP/μl from healthy controls, and RRMS patients (see Figure 6B). Discontinuous line marks the average resistance of TNF-stimulated cells. (PDF 269 KB) [file 12868_2014_3798_MOESM1_ESM.pdf]

SUPPLEMENTARY INFORMATION

Microparticles in multiple sclerosis and clinically isolated syndrome: effect on endothelial barrier function

Beatriz Marcos-Ramiro et al.

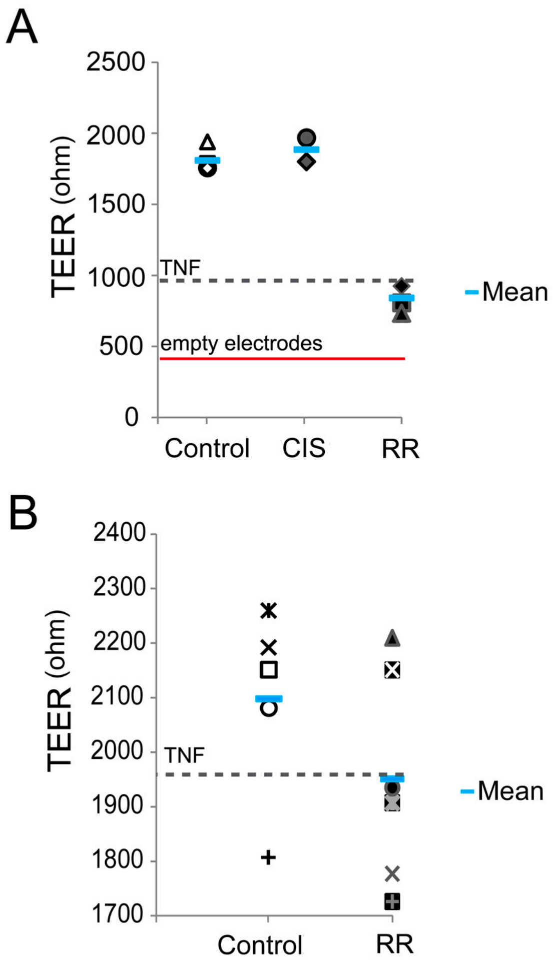

Supplementary Figure 1: Absolute values of TEER detected in endothelial cell monolayers exposed for 14 h to MPs from healthy controls and patients. (**A**) TEER of HUVECs incubated with 1000 MP/µl from healthy controls, CIS and RRMS (RR) patients (see Figure 5D). Red line marks the average resistance detected in empty electrodes. (**B**) TEER of HCMEC/D3 incubated with 2000 MP/µl from healthy controls, and RRMS patients (see Figure 6B). Discontinuous line marks the average resistance of TNF-stimulated cells.
